# Trends in levels of self-reported psychological distress among individuals who seek psychiatric services over eight years: a comparison between age groups in three population surveys in Stockholm County

**DOI:** 10.1186/s12888-017-1499-4

**Published:** 2017-10-11

**Authors:** Kyriaki Kosidou, Andreas Lundin, Glyn Lewis, Peeter Fredlund, Henrik Dal, Christina Dalman

**Affiliations:** 1 Centre for Epidemiology and Community Medicine, Stockholm County Council, Solnavägen 1 E, -113 65 Stockholm, SE Sweden; 2 Department of Public Health Sciences, Karolinska Institutet, Solnavägen 1 E, -113 65s Stockholm, SE Sweden; 3 Division of Psychiatry, University College London, Maple House, 149 Tottenham Court Rd, London, W1T 7NF UK

**Keywords:** Trends, Psychological distress, Psychiatric service use, Young adults, Help-seeking

## Abstract

**Background:**

Psychiatric service use has increased in Sweden and in other developed countries, particularly among young people. Possible explanations include lower threshold for help-seeking among young people, but evidence is scarce.

**Methods:**

We analysed the 2002, 2006 and 2010 Stockholm public health surveys for changes in the mean level of psychological distress among adult users of psychiatric in- and outpatient services in four age groups: 18–24, 25–44, 45–64 and ≥65 years. Psychological distress was measured via the 12-item General Health Questionnaire (GHQ-12), using the Likert scoring method 0–1–2-3. In- and out-patient psychiatric service use within 6 months from the surveys was obtained from registers.

**Results:**

The mean level of distress among young adults 18–24 years who utilize psychiatric services decreased between 2002 (mean GHQ-12 score, 95% confidence interval 20.5, 18.1–23.0) and 2010 (16.2, 14.6–17.7), while it remained fairly stable in older age groups. Results were similar in sex-stratified analyses, although the decrease was statistically significant only among young women 18–24 years. At the end of the follow-up, the level of distress among patients was similar for all age-groups.

**Conclusions:**

There were no differences between age groups in the level of distress when seeking care at the end of the follow-up period, supporting that there is no age-specific over- or under-consumption of psychiatric care in later years. However, the lowered threshold for help-seeking among young adults over time might have contributed to increases in psychiatric service use in the young age group. Public health policy and service delivery planning should consider the needs of the widening group of young users of psychiatric services.

## Background

The use of mental health services appears to have increased in Sweden and in other developed countries, particularly among young people [1–8]. In Sweden, mental health service use has increased sharply among children (<18 years) and young adults (≤29 years) while remained stable or showed smaller increases in older age groups since the 1990ies [3, 4]. Studies from USA also report sharper increases in mental health service use among the young (<20 years) compared to older age groups during the past decades [1, 2, 8].

It could be hypothesized that increased prevalence of mental ill-health explains increases in service use. Many studies have, indeed, reported increasing long-term trends in self-reported psychological distress and self-harm among young people in developed countries since the 1980s [3, 5, 9–13]. However, several population-based studies on the prevalence of clinically significant depression and anxiety indicate stable levels of these disorders during recent years [14, 15].

Although psychiatric morbidity is a strong predictor of mental health service use, factors such as the social acceptability of mental illness, awareness about mental health problems, access to mental health services, increasing adherence to clinical practice standards by mental care providers, as well as a broadening conceptualization of mental illness and the perceived need for care are also important, as discussed by PLOS Medicine editors [16] and supported by several studies [17–19]. All of these factors may have increased help-seeking for mental health problems also among less severe cases, which might explain increases in service use. In this regard, the characteristics of the widening group of users of psychiatric services could provide some important insights. For example, reduced symptom severity among individuals who seek mental health care would imply a lower threshold for help-seeking in recent years. Yet, no population-based study has examined levels of psychological distress among users of psychiatric services in different age groups and whether there have been changes over time. Some studies [20, 21] have indicated lower threshold for seeking mental health care among children. There is a need to understand these patterns among adults in order to inform policy makers and service delivery planners about the healthcare needs of the population.

Here we examine whether there are differences between age groups in their levels of distress when seeking care, and if these patterns change over time. We examine levels of self-reported psychological distress in relation to psychiatric in-patient and outpatient service use among young adults as compared to older age groups. We utilize three large population-based representative samples from Stockholm County, Sweden, collected four and eight years apart.

## Methods

### Study population

We used data from the Stockholm Public Health Surveys in the years 2002 (*n* = 23,771), 2006 (*n* = 34,667) and 2010 (*n* = 30,730) [22]. The sampling frame consisted of residents of Stockholm County who were listed in the Swedish Total Population Register. For each survey, an area-stratified random sample of adults aged 18–84 years (2002, 2006) or 18 years and older (2010) was invited to complete self-administered questionnaires assessing a variety of health, lifestyle and social characteristics. A complete description of the surveys and the available questionnaire-based data has been published elsewhere [22]. In total *N* = 49,909, *N* = 56,634, and *N* = 55,341 individuals were invited to participate in 2002, 2006 and 2010, respectively. Response rates were 62.5% in 2002, 61.5% in 2006 and 55.6% in 2010. Participants in the 2002 survey were followed up with a new questionnaire in 2007 when they were asked for informed consent. The 2002 subsample comprises only those participants in the 2002 survey who provided informed consent in the 2007 follow-up (2002–2007 retention rate 76%). Non-responders were more likely to be men, born outside Sweden, under age 45, single or separated, and unemployed or low-income [22]. The surveys assessed psychological distress using the 12-item General Health Questionnaire (GHQ-12) [23]. As all Swedish residents receive a unique personal identification number at birth or upon obtaining a residency permit, we were able to link respondents of the Stockholm Public Health Surveys to a range of administrative and health care registers. We were, thus, able to identify survey respondents that had prospectively used psychiatric care within six months following each survey. In total there were 2276 participants in the Stockholm Public Health Surveys who used psychiatric services, including 538 participants from the 2002 survey, 920 from the 2006 survey and 818 from the 2010 survey, who comprise our study population. Individuals with missing data on GHQ-12 (*n* = 31) were excluded from the final analytical sample (*N* = 2245).

### Psychological distress

Psychological distress was assessed using the 12-item version of the General Health Questionnaire (GHQ-12) [23], which has been validated for use in the Swedish population [24, 25]. Item scores were coded according to the Likert method (0–1–2-3) [26] and a total GHQ-12 score ranging from 0 to 36 was computed. Internal missing was small, with 1.4% who did not complete the entire GHQ scale.

### Psychiatric service use

Data on psychiatric service use was collected from the Information and Statistics Division, Stockholm County Council, which collates a centralized database including all mental health service utilization. From this database, we obtained all inpatient episodes and all out-patient contacts at psychiatric clinics, including visits to private psychiatrists contracted by Stockholm County Council, in Stockholm County within 6 months of participating in each survey.

### Covariates

Information on age and sex were obtained from administrative registers. We categorized age as: 18–24, 25–44, 45–64, and 65 years or older. We supplemented the dataset with information from the Longitudinal Integration Database for Health Insurance and Labour Market Studies (Swedish acronym LISA). Potential confounders included country of birth (coded as Sweden or other), level of educational attainment (compulsory, upper secondary, higher education), household disposable income quartiles, and being gainfully employed (Yes/No). Internal missing was small and limited to education (2.9%).

### Ethical considerations

Written informed consent was obtained from all the study participants and ethical approval for the study was granted by the Stockholm Regional Ethical Review Board.

### Data analyses

All analyses were carried out in the statistical software SAS v. 9.3 (SAS Institute Inc. Cary, NC, USA). We performed regression analyses using dummy variables. Since the data were collected with area-stratified random samples, we used the SURVEYREG procedure in SAS 9.3 to calculate adjusted least square means and confidence intervals (CIs). We compared mean GHQ-12 scores and 95% CIs for participants in different age groups (18–24, 25–44, 45–64, and 65 years or older) who used psychiatric services. F-tests were used for trend analysis over time. Characteristics such as sex, country of birth, education, household disposable income and having gainful employment were adjusted for. We also examined results in a sex-stratified model. In sensitivity analyses, regression analyses were repeated for psychiatric service use within a shorter follow-up time of two months.

Weights were used to correct for the stratified random sample design as well as systematic non-response. Statistics Sweden provided the non-response calibration weights on the basis of available auxiliary variables from national registries, their association with selected variables from the surveys and association with probability of participating in the survey. The auxiliary variables included sex, age, country of birth, civil status, income, educational level, sickness allowance and area of residence [27].

## Results

There was an increase in overall psychiatric service use progressively with each wave of survey, such that the proportion of survey participants using psychiatric services increased from 2.5% in 2002 to 2.9% and 3.2% in 2006 and 2010, respectively (F = 7.58, *p* = 0.0005) (Table 1).

**Table 1:** Characteristics of participants in the 2002, 2006 and 2010 Stockholm Public Health Surveys

	2002 survey		2006 survey		2010 survey	
	% weighted sample		% weighted sample		% weighted sample	
	All, *n* = 23, 771	Psychiatric service users^a^, *n* = 538 (2.5% of survey n)	All, *n* = 34,667	Psychiatric service users^a^, *n* = 920 (2.9% of survey n)	All, *n* = 30,730	Psychiatric service users^a^, *n* = 818 (3,2% of survey n)
Women	51.3	65.3	50.9	65.3	51.2	62.7
Age group						
18–24 years	12.1	15.8	10.8	14.2	12.0	21.2
25–44 years	41.4	54.2	40.0	45.4	36.9	42.6
45–64 years	32.6	26.2	33.3	33.9	31.3	29.6
65+ years	13.9	3.9	15.9	6.5	19.8	6.7
Born outside Sweden	23.1	23.2	22.9	26.3	24.6	21.7
Education						
Compulsory	20.6	22.8	20.9	21.9	21.9	28.3
Upper secondary	43.9	42.8	41.4	43.9	39.7	38.5
Higher	35.5	34.4	37.7	34.2	38.5	33.2
Income						
Lower quartile	30.5	44.2	30.4	43.6	30.4	38.4
2nd quartile	24.3	27.5	23.7	26.2	24.2	33.2
3^d^ quartile	22.9	17.9	22.8	18.3	23.4	18.5
Highest quartile	22.3	10.4	23.1	12.00	22.0	10.0
Being gainfully employed	70.2	54.7	63.2	43.8	62.7	45.9
Mean GHQ-12 Likert score by age group						
18–24 years	11.4	19.7	11.0	16.7	10.6	15.2
25–44 years	11.1	17.2	10.5	16.3	10.1	16.0
45–64 years	10.4	15.3	10.0	15.3	9.9	16.2
65+ years	9.5	13.0	9.4	13.5	9.3	12.7

^a^Psychiatric service use within 6 months from the survey

Table 1 also shows the prevalence of covariates (unweighted and weighted) among all survey participants and the patient subgroup. Women, participants with lower income, and those without gainful employment, were more likely to use psychiatric services. Among the patients, there seemed to be a relative shift in age-groups between the 2000 and 2010 surveys, with 18–24 year olds becoming a proportionately larger group in psychiatric care. There was no increase in mean GHQ scores in the entire survey population across the three surveys. In the patient group, there was a higher mean GHQ score (unadjusted) among the young compared to older age groups in 2002, which declined over time.

**Fig. 1:**
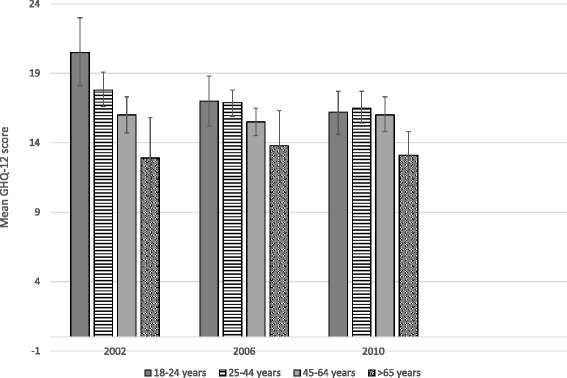
Mean levels of psychological distress among participants in the Stockholm Public Health Surveys who use psychiatric services, in 2002, 2006 and 2010

Table 2 and fig. 1 show the adjusted mean levels of psychological distress across age groups among psychiatric service users (*n* = 2245). Adjustment for confounders seemed to slightly increase estimates of mean levels of distress among patients. In 2002, those in the young age group (18–24 years) seeking psychiatric care appeared to have higher levels of distress as compared to age groups older than 44 years (*p* < 0.001 for comparison to both the 45–64 and the 65+ years age groups). This was true also for those aged 25–44 years (*p* < 0.05 for comparison to both the 45–64 and the 65+ years age groups). However, mean levels of distress among young patients (18–24 years) decreased significantly between 2002 and 2010. This trend was also discernible among those 25–44 years old, resulting in similar mean levels of distress among those seeking psychiatric care at end of follow-up (year 2010) for those under 65 years. Patients older than 65 years appeared to have a lower mean level of self-reported distress overall years. Formal test of interaction between age groups and surveys found no statistical evidence of an interaction in terms of probability of service use (*p* = 0.229).

**Table 2:** Mean level of psychological distress among users of psychiatric services in different age groups (*n* = 2245)

	Year of survey			
	2002	2006	2010	
	Mean GHQ-12 score^a^, 95% CI			F-test for trend 2002–2010
Age group				
18–24 years	20.5 (18.1–23.0)	17.0 (15.2–18.8)	16.2 (14.6–17.7)	*p* = 0.005
25–44 years	17.8 (16.6–19.1)	16.9 (15.9–17.8)	16.5 (15.4–17.7)	*p* = 0.22
45–64 years	16.0 (14.7–17.3)	15.5 (14.5–16.5)	16.0 (14.8–17.3)	*p* = 0.69
65+ years	12.9 (10.1–15.8)	13.8 (11.3–16.3)	13.1 (11.3–14.8)	*p* = 0.78

^a^Adjusted for sex, country of birth, education, household disposable income and being gainfully employed

Tables 3 and 4 show results of sex-stratified analyses. There was a decreasing trend in mean levels of distress among male patients up to 44 years of age, however, it was not statistically significant. No clear pattern was found among male patients in older age groups (Table 3). Among female patients, there was a clear downward trend in mean levels of distress in the young age group (18–24 years), while no such trend was found in older age groups (Table 4).

**Table 3:** Mean level of psychological distress among male users of psychiatric services (*n* = 690)

	Year of survey			
	2002	2006	2010	
	Mean GHQ-12 score^a^, 95% CI			F-test for trend 2002–2010
Age group				
18–24 years	21.9 (17.7–26.2)	18.0 (15.5–20.5)	17.5 (14.6–20.3)	*p* = 0.13
25–44 years	18.6 (16.3–21.0)	16.9 (15.4–18.3)	15.8 (14.0–17.6)	*p* = 0.12
45–64 years	15.7 (13.7–17.7)	15.7 (13.8–17.6)	14.2 (12.3–16.2)	*p* = 0.41
65+ years	13.8 (11.5–16.0)	14.3 (10.1–18.4)	15.9 (12.4–19.4)	*p* = 0.49

^a^Adjusted for country of birth, education, household disposable income and being gainfully employed

**Table 4:** Mean level of psychological distress among female users of psychiatric services (*n* = 1586)

	Year of survey			
	2002	2006	2010	
	Mean GHQ-12 score^a^, 95% CI			F-test for trend 2002–2010
Age group				
18–24 years	20.1 (17.4–22.9)	16.8 (14.4–19.1)	15.7 (13.9–17.6)	*p* = 0.04
25–44 years	18.1 (16.7–19.4)	17.2 (16.1–18.4)	17.4 (15.9–18.9)	*p* = 0.65
45–64 years	16.8 (15.0–18.5)	15.6 (14.5–16.7)	17.6 (16.1–19.1)	*p* = 0.08
65+ years	12.6 (8.0–17.2)	13.7 (10.6–16.8)	12.5 (10.6–14.3)	*p* = 0.69

^a^Adjusted for country of birth, education, household disposable income and being gainfully employed

In sensitivity analyses, the pattern of changes in mean levels of psychological distress among adults in different age groups who used psychiatric services within two months from the surveys was similar to that for service use up to six months from the surveys (data not shown).

## Discussion

This study examines trends in levels of psychological distress among individuals who use psychiatric services in different age groups in the general adult population of Stockholm County, Sweden, over a period of eight years. At the beginning of the study period in 2002, young adults ages 18–24, and to some extent also those 25–44 years old, who utilized psychiatric services, appeared to have higher level of distress as compared to older age groups. However, in contrast to patients in older age groups who underwent little change in mean levels of psychological distress between 2002 and 2010, younger patients had a decrease in mean level of distress during this period, although only significant for the youngest (18–24 years of age). At the end of the study period in 2010, there were no longer differences in mean levels of distress among patients of working age (18–64 years). Mean population levels of psychological distress remained stable or decreased while psychiatric service use increased during the study period, indicating that factors other than increased prevalence of psychiatric morbidity might explain increases in service use. Our findings indicate that this increase in service use might partly be explained by a lowering of threshold for seeking mental health services among young people in Sweden during the first decade of the twenty-first century and that there are no specific patterns of under- or overconsumption in any age-group at the end of follow-up period.

Our finding of a decrease in mean level of psychological distress among young adults who use psychiatric services corroborates findings in previous studies among children and adolescents. A study from USA [1] that examined trends in mental health service use in youth between 1996 and 1998 and 2010–2012 found that youth with less severe or no mental health impairment, measured by parental reports, accounted for most of the absolute increases in service use. A study from Finland found increases in the parental perceived need for mental health care and mental health service utilization among children, but stable levels in children’s mental health problems since the 1990s [18]. Their results indicated decreasing long-term trends in the parental threshold for seeking mental health services for children in Finland. Lastly, a recent study from Great Britain found that while parental and teacher reports of children’s mental health problems decreased during the first decade of the twenty-first century, the teacher- and parental- perceived impact of such problems increased during the same period [19].

One limitation of previous literature is that parental reports have shown to be less valid than self-reports in rating mental health problems in children and adolescents [26, 27]. Our study is the first to assess self-reported psychological distress in relation to service utilization among adult age groups, and adds that average severity of symptoms in young adults utilizing psychiatric services has decreased during the first decade of the twenty-first century. Taken together, these results support the notion that there has been a lowering of threshold for seeking psychiatric services among younger age groups.

A possible explanation for a lower threshold of help seeking is that the perceived need for mental health care has increased among young people in these countries. Young people in developed countries are facing rising educational demands and competitive job markets, as well as an increasing range of potential social roles and opportunities. Such challenging prospects may generate stronger expectations regarding an individual’s level of functioning and increase the perceived need for mental health care. In addition, broader conceptualization of mental illness and increased accessibility of mental health services may have enhanced help-seeking.

Furthermore, increased societal emphasis on young peoples' psychosocial health and awareness of the importance of early recognition and treatment of psychiatric problems might have improved young peoples´ attitude towards use of psychiatric services. Previous studies have shown young people to be reluctant to seek mental health care and efforts have been put to improve mental health and service utilization in the young age group [28, 29]. We found that while young adults who utilized psychiatric services had a higher level of psychological distress at the beginning of the study period, compared to older adults, there was no difference in mean levels of distress among patients in working-age age groups eight years later. These findings support the notion that young people were previously underrepresented within psychiatric care services in relation to their level of mental ill-health, compared to other age groups, and that this has been eliminated during recent years.

It remains to be known how much a previous underrepresentation of young people within psychiatric services, or an increase in the perceived need for care in the young age group, have contributed to our results. Nevertheless, our study clearly shows that there has been a relative shift in age groups among psychiatric patients in Stockholm County, with young adults becoming a relatively larger patient group.

Thus, our results have implications for service planning and policies regarding provision of mental health care. There seems to be a widening group of young users of psychiatric services and the needs as well as mental health and social outcomes in this group merit further research. The use of psychiatric services at a lower threshold in young adults emphasizes the need to improve mental health services for young people outside specialised psychiatric clinics as well as to enhance mental well-being for those in the young age group. This is also, of course, valid for older age groups, who, at the end of our study, had similar levels of distress at care seeking. Lastly, despite increases in service use, still only a proportion of individuals with mental disorders seek mental health treatment, regardless of age group [30]. Thus, health services should continuously seek to improve access to mental health care.

The large population-based samples collected over a period of eight years and the combined use of self-reported and register-based data are some strengths of this study. Attrition is a limitation of the study, albeit accounted for, to some extent, by calibration weights. The GHQ-12 has been shown to measure psychological distress regardless of age [21, 31], but the stability of levels of distress over time has not been tested in different age groups. If chronicity of distress differs between age groups, that may have influenced levels of service use within six months in our study. However, results of sensitivity analyses revealed a similar pattern of change in levels of psychological distress among patients that used psychiatric services up to two months from the surveys as compared to six months, though statistical power was limited. Another limitation is that we measure psychological distress with a self-report questionnaire, which implies imperfect sensitivity and specificity to psychiatric morbidity compared with diagnostic interviews. However, this imprecision is likely to be similar across the surveys and would not affect the trend. Previous studies have shown psychological distress measured by the GHQ to be an adequate index of psychiatric morbidity and predictive of impaired functioning and mental health care utilization [22, 23, 32], Lastly, we did not have information on care for mental health problems in primary care, which is a limitation of our study.

## Conclusions

In conclusion, we found a lowering of mean level of psychological distress among young adults in the population that use psychiatric services during the first decade of the twenty-first century. Thus, a lower threshold for help-seeking among young people might explain the recent increase in psychiatric service use in Sweden and perhaps in other developed countries. Yet, at end of follow-up there were no differences between age-groups in level of distress among those seeking care, supporting that there is no age specific over/under consumption of psychiatric care in later years. The needs of the widening group of young users of psychiatric services merit further research.
